# Chronic cluster headache and the pituitary gland

**DOI:** 10.1186/s10194-016-0614-0

**Published:** 2016-03-11

**Authors:** Annelien De Pue, Bart Lutin, Koen Paemeleire

**Affiliations:** Department of Neurology, Ghent University Hospital, De Pintelaan 185, B-9000 Ghent, Belgium; Department of Radiology, Ghent University Hospital, Ghent, Belgium

**Keywords:** Cluster headache, Pituitary gland, Secondary headache

## Abstract

**Background:**

Cluster headache is classified as a primary headache by definition not caused by an underlying pathology. However, symptomatic cases of otherwise typical cluster headache have been reported.

**Case presentation:**

A 47-year-old male suffered from primary chronic cluster headache (CCH, ICHD-3 beta criteria fulfilled) since the age of 35 years. A magnetic resonance imaging (MRI) study of the brain in 2006 came back normal. He tried several prophylactic treatments but was never longer than 1 month without attacks. He was under chronic treatment with verapamil with only a limited effect on the attack frequency. Subcutaneous sumatriptan 6 mg injections were very effective in aborting attacks. By February 2014 the patient developed a continuous interictal pain ipsilateral to the right-sided cluster headache attacks. An indomethacin test (up to 225 mg/day orally) was negative. Because of the change in headache pattern we performed a new brain MRI, which showed a cystic structure in the pituitary gland. The differential diagnosis was between a Rathke cleft cyst and a cystic adenoma. Pituitary function tests showed an elevated serum prolactin level. A dopamine agonist (cabergoline) was started and the headache subsided completely. Potential pathophysiological mechanisms of pituitary tumor-associated headache are discussed.

**Conclusion:**

Neuroimaging should be considered in all patients with CCH, especially those with an atypical presentation or evolution. Response to acute treatment does not exclude a secondary form of cluster headache. There may be shared pathophysiological mechanisms of primary and secondary cluster headache.

## Background

Cluster headache (CH) is classified as a primary headache by definition not caused by an underlying pathology. However, numerous symptomatic cases of otherwise typical CH have been reported. We want to report a case of a man with typical chronic cluster headache (CCH) and a pituitary lesion only found on repeat MRI. Potential mechanisms underlying this association are discussed. We hope this will be useful to other clinicians taking care of patients suffering from this devastating condition [1].

## Case presentation

**Fig. 1:**
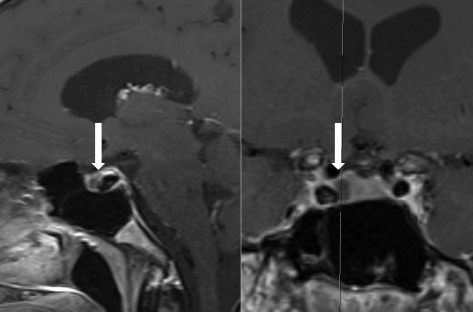
MRI of the pituitary gland. T1 contrast-enhanced images. *Left* image: sagittal view. *Right* image: coronal view. *White* arrow indicates cystic structure in the pituitary

A 47 year-old man was diagnosed with CH at the age of 35. A 1,5 Tesla MRI of the brain in another hospital was reportedly normal. The patient came under our care in 2011. The headache attacks and pattern were compatible with a primary CCH diagnosis according to the criteria of the International Headache Society (ICHD-3 beta, 3.1.2) [2]. The patient described attacks of strictly right-sided orbitotemporal headache associated with ipsilateral tearing and nasal congestion. The attacks lasted from 20 to 60 min, with a frequency up to 4 attacks per day, often including one attack at night. The most consistent trigger was alcohol intake. Subcutaneous sumatriptan 6 mg injections were very effective in aborting attacks, inhaled high-flow oxygen was not. Despite different prophylactic treatments (verapamil up to 480 mg/day, lithium up to 800 mg/day, topiramate up to 400 mg/day) the patient was never longer than 1 month without attacks. By February 2014 the patient developed a continuous interictal pain ipsilateral to the right-sided CH attacks. At that point in time he was under chronic treatment with verapamil (maximal tolerated dose of 560 mg/day) for 6 months with only a mild effect on attack frequency. An indomethacin test (up to 225 mg/day orally) was negative. Because of the change in headache pattern we decided to perform a new brain MRI, which showed a cystic structure in the pituitary gland (Fig. 1). The differential diagnosis was between a Rathke’s cleft cyst or a cystic adenoma. Pituitary function tests (PFTs) came back normal, except for an elevated prolactin level (68.4 μg/L, normal values 4–17 μg/L) and a low free testosterone level (4.44 ng/dL, normal values 6–25 ng/dL). This could be due to verapamil-induced hyperprolactinemia or a prolactinoma. There was no galactorrhea, gynaecomastia, or erectile dysfunction (but he acknowledged a low libido), and visual fields were full. The patient was not willing to stop the treatment with verapamil as he feared an increase in cluster attack frequency. The patient was referred to an endocrinologist, who started carbergoline, a dopamine agonist, at a dose of 0,25 mg twice a week. Within a few weeks after the start of the cabergoline treatment the CH attacks subsided completely and verapamil could be successfully stopped. The serum prolactin and testosterone levels normalized. A repeat MRI in October 2015 demonstrated a 30 % reduction in size of the cystic pituitary lesion. At present the patient has been on carbergoline treatment for 1,5 year and the cluster headaches haven’t returned ever since and prolactin levels remained within normal range. Interestingly, the patient reports to experience a new type of unilateral frontotemporal headache attacks since about 6 months. These attacks are milder and shortlasting (seconds), and the pain is rather stabbing. There are no associated cranial autonomic symptoms. These attacks are not disabling and the patient does not need treatment for them. We have labeled these probable short-lasting unilateral neuralgiform headache attacks (ICHDI-3 beta, 3.5.3) within the given context.

## Discussion

### Secondary cluster headache

The lesion in the pituitary and the headaches could just be a co-occurrence. However the temporal relationship between the initiation of carbergoline treatment and disappearance of the cluster headache attacks suggests a potential causal relationship with the pituitary lesion and (worsening of) the headache condition. This adds to numerous recent reports of secondary cluster-like headache (CLH). By definition before concluding to a primary headache potential causative disorders should be excluded to rule out a secondary CLH. Edvardsson reviewed 63 cases of symptomatic CH associated with vascular problems (44 %), tumours (40 %) or inflammation/infection (11 %) [3]. Of the 63 cases 48 % fulfilled the criteria for CH. A larger cohort of 156 cases of CLH showed a similar distribution of causative disorders [4]. On first observation, 50 % of CLH perfectly mimicked CH at presentation [5]. Furthermore, the response to typical CH medications does not exclude a secondary form. Red flags could be older age at onset, abnormalduration/frequency/localization, change in clinical characteristics or response to treatment, or an abnormal neurological/general examination [4, 5]. These findings suggest that neuroimaging should be considered in all patients with CH, even typical cases. The ICHDIII beta criteria for secondary headache disorders require evidence of causation, which includes that ‘headache has significantly worsened in parallel with worsening of the presumed causative disorder’ and ‘headache has significantly improved (or disappeared) in parallel with improvement of the presumed causative disorder’. In our patient neuro-imaging was done early in his headache trajectory and results came back normal. However, in retrospect we noted that the spatial resolution of the initial MRI of the brain at the level of the pituitary gland was too low to exclude that the cystic structure (Fig. 1) was already present. Even though interictal headache in cluster headache is not an uncommon phenomenon [6], it was the change in headache history in our patient that sparked repeat neuro-imaging.

### Pathophysiology

**Fig. 2:**
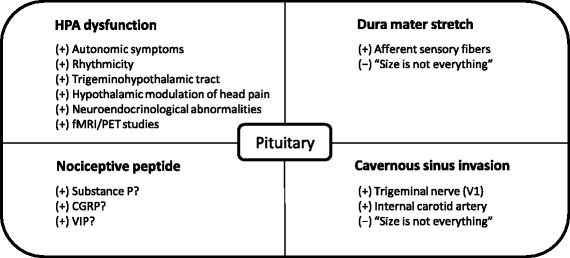
Potential pathophysiological links with the pituitary gland in cluster headache. (+) arguments pro (-) arguments con. “Size is not everything” is a reference to an article of Levy et al. [16]. V1 is the ophthalmic branch of the trigeminal nerve. HPA = hypothalamic-pituitary axis. The figure is further explained in the text under the heading “pathophysiology”

The pathophysiology of secondary CLH associated with pituitary disorders is not well-known. A few hypotheses are considered and summarized in Fig. 2. The long-held theory that CH was the related to a cerebrovascular problem radically changed in the late nineties with CH attacks being recoined as neurovascular in origin and with an important role for the hypothalamic-pituitary axis in the generation of these attacks. Functional imaging studies (fMRI, PET), neuro-endocrine changes (melatonin, cortisol, testosterone, …) and the circadian/circannual rhythmicity all point to an alteration in the hypothalamus [7–10]. Also lithium, which is one of the known preventative treatment options for CH, is heterogeneously distributed in the brain and seems to accumulate in the hypothalamus and pituitary gland [11, 12]. The pain during cluster headache attacks is attributed to activation of the trigeminovascular system and cranial autonomic symptoms are generated via the trigeminal-autonomic reflex [13–15].

A structural lesion in the hypothalamic-pituitary axis could lead to an autonomic imbalance resulting in an attack-wise presentation of complaints. Others have suggested that the headache due to a lesion in the pituitary is a result of dura mater stretch or invasion of the cavernous sinus. Indeed, the cavernous sinus lateral to the sella turcica contains the ophthalmic and maxillary branches of the trigeminal nerve as well as the internal carotid artery, which are structures that can generate pain. However, in a systematic study of headache in patients with pituitary tumors, no correlation between pituitary volume and headache or between headache and cavernous sinus invasion was found [16]. Pituitary tumor-associated headache may have a biochemical-neuroendocrine basis rather than a structural one [16]. Finally, there is some evidence for the role of “nociceptive peptides” [17]. The presence of calcitonin gene related peptide (CGRP) or substance P in pituitary tumors does not seem to be associated with headache [15]. Other candidate peptides are vasoactive intestinal peptide (VIP), pituitary adenylate cyclase-activating protein and neuropeptide Y.

### Pituitary function tests, prolactin and dopamine agonist

A few additional questions are provoked by this case. Is the raised serum prolactin level due to the lesion in the pituitary or induced by the high dose of verapamil? Is the effect of cabergoline proving that the lesion is a prolactinoma and the cause of the headache? Is the positive effect of carbergoline due to the normalization of serum prolactin? Is there a need for screening with PFT’s in CH?

There are physiologic, pituitary and systemic (including medications, such as verapamil) causes of hyperprolactinemia [18]. Pituitary causes include prolactin-secreting pituitary adenoma or disconnection hyperprolactinemia due to a lesion that compresses the pituitary stalk [18]. L-type calcium channel blockers, such as verapamil, are known to cause a doubling of serum prolactin levels [19]. Since the patient was reluctant to stop verapamil because of fear increased CH attack frequency, we could not distinguish between both mechanisms prior to initiation of cabergoline treatment. Cabergoline is a long acting D2 dopamine receptor agonist that inhibits prolactin secretion. There are reported cases of microprolactinomas manifesting with headache that resolved after administration of a dopamine agonist [20, 21], but there are also reports stating the opposite [22]. The effect of cabergoline on headache doesn’t seem to be associated with the normalization of serum prolactin [20]. Potential mechanisms include alterations to the pain-modulating dopaminergic system and carbergoline, an ergot derivative, also possesses significant affinity for certain subtypes of serotonergic and adrenergic receptors. A normal MRI of the brain does not exclude a microadenoma [23], and PFT’s should therefore be considered in (refractory) patients with CCH or other (TAC).

## Conclusion

Neuroimaging should be considered in all patients with CCH, especially those with an atypical presentation or evolution. When performing brain imaging it seems important to pay extra attention to the pituitary/parasellar region. Response to acute treatment does not exclude a secondary form of CH. PFTs should be considered in patients suffering from (refractory) CCH or other TACs. Cabergoline may have a dramatic effect on CH in patients with coexistent hyperprolactinemia.

### Consent

The patient gave written informed consent to have his case published.

## Abbreviations

CCH: chronic cluster headache
CGRP: calcitonin gene-related peptide
CH: cluster headache
CLH: cluster-like headache
ICHD-3 beta: The International Classification of Headache Disorders Third Edition, beta version
mg: milligram
MRI: magnetic resonance imaging
ng/dl: nanogram per deciliter
PFT: pituitary function tests
TAC: trigeminal autonomic cephalalgias
TCC: trigeminocervical complex
VIP: vasoactive intestinal peptide
μg/l: microgram per liter
